# Gut Microbiome Contributes to Liver Fibrosis Impact on T Cell Receptor Immune Repertoire

**DOI:** 10.3389/fmicb.2020.571847

**Published:** 2020-11-27

**Authors:** Qing Liang, Meina Zhang, Yudi Hu, Wei Zhang, Ping Zhu, Yujie Chen, Pengxin Xue, Qiyuan Li, Kejia Wang

**Affiliations:** ^1^National Institute for Data Science in Health and Medicine, School of Medicine, Xiamen University, Xiamen, China; ^2^Department of Pathology, The 971 Hospital of People’s Liberation Army Navy, Qingdao, China; ^3^Department of Gynecology and Obstetrics, The 971 Hospital of People’s Liberation Army Navy, Qingdao, China

**Keywords:** gut microbiome, T cell receptor, immune repertoire, liver fibrosis, immune microenvironment

## Abstract

Gut microbiota (GM) modifies the intrahepatic immune microenvironment, but the underlying mechanisms remain poorly understood. Liver fibrosis-associated imprinting is predicted to be reflected in GM. This study investigated the link between GM and the intrahepatic T cell receptor (TCR) immune repertoire (IR), and whether GM modulates the intrahepatic immune microenvironment via TCR IR during liver fibrosis. We analyzed the correlation between GM and TCR IR during liver fibrogenesis. Accordingly, 16S rRNA gene sequencing (16S-seq) and bulk immune repertoire sequencing (IR-seq) were performed to characterize GM and intrahepatic TCR IR. Fecal microbial transplant (FMT) and TCRβ knockout (Tcrb^KO^) mouse models were employed to determine the biological link between GM and TCR IR in liver fibrosis. We found that GM and intrahepatic TCR IR are highly correlated, with both showing reduced diversity and centralized distribution during liver fibrosis. The restoration of normal intestinal microbiota may reshape intrahepatic TCR IR and delay liver fibrosis. Interestingly, TCR IR ablation abrogated the impact of GM on liver fibrogenesis. Furthermore, GM modulated hepatic stellate cell (HSC) activation via TCR IR-mediated intrahepatic immune milieu. Our study demonstrates that GM, which exhibits cross-talk with the intrahepatic TCR IR, influences the intrahepatic immune microenvironment and liver fibrosis progression.

## Introduction

The gut microbiota (GM) (comprising more than 100 trillion bacteria) represents a “footprint” of genetic and environmental factors. The GM has been implicated in the pathophysiology of many intestinal and extraintestinal diseases (Tilg et al., 2016). GM, considered a virtual organ, forms axes with various extraintestinal organs, including the brain, liver, and kidney as well as cardiovascular and endocrine systems. Characteristically, the gut-liver axis is a close anatomical, functional, and bidirectional interaction that occurs primarily through the portal circulation (Milosevic et al., 2019). Intestinal antigens (IAg) (originating either from pathogenic microorganisms or from food) that enter the portal circulation, are captured by intrahepatic antigen-presenting cells (APCs), leading to T cell receptor (TCR) recognition and hence the activation of the adaptive immune system. The interaction between the GM and intrahepatic immune microenvironment is regulated and stabilized by a complex network of factors including IAg, immune cells, and cytokines. GM dysbiosis leads to the excessive production of bacterial fragments and products [including lipopolysaccharides (LPS), peptidoglycans, and flagellin], which reach the liver through the portal system, contributing to chronic inflammation and liver fibrosis (Zhou et al., 2019). Emerging evidence suggests that the GM is altered following intestinal microbiota transplantation in patients with pre-cirrhotic liver disease and cirrhosis (Ohtani and Kawada, 2019; Bajaj and Khoruts, 2020). Liver fibrogenesis triggers an upsurge in the number of bacteria in circulation, indicating that intestinal microbiota modify the progression of liver fibrosis (Tilg et al., 2016).

The liver is structurally and functionally heterogeneous and is known to induce immune tolerance and immunity (Breous et al., 2009). In the liver, cell types such as hepatocytes, immune cells, and hepatic stellate cells (HSC) interact with each other. Recent studies have investigated the crucial role of different intrahepatic immunocyte subsets (including T cells, B cells, neutrophils, macrophages, and innate lymphoid cells) in the progression of liver inflammation and fibrosis (Koyama and Brenner, 2017; Tosello-Trampont et al., 2017; Wahid et al., 2018). An aberrant intrahepatic immune microenvironment (aberrant immune cell distribution and cytokines/chemokine secretion) induces HSC transdifferentiation from quiescent lipocytes into myofibroblast-like cells to drive fibrogenesis (Friedman, 2008; Higashi et al., 2017). Evidence from studies indicates that adaptive immune cells, especially T and B cells, can modulate inflammation and fibrosis in response to liver injury (Novobrantseva et al., 2005; Holt et al., 2006; Hammerich et al., 2014). However, the links between GM and the adaptive immune system in the pathogenic progression of liver fibrosis and whether GM modulates HSC activation in the intrahepatic immune microenvironment under the context of chronic liver injury remain largely unknown.

T cells, expressing a TCR heterodimer consisting of αβ or γδ chains, belong to the T lymphocyte subset. The complementarity-determining region 3 (CDR3) region of TCRβ plays a critical role in the major histocompatibility complex (MHC)-peptide complex recognition, which determines antigen recognition, immune activation, clonal expansion, and selection for T cells (Pannetier et al., 1993; Nikolich-Zugich et al., 2004). The CDR3 region is composed of variable (V), diversity (D), and the joining (J) domains spanning from the terminus of the V domain to the beginning of the J domain. It is thought that the hypervariable CDR3 region of TCRβ, referred to as the TCR IR, mediates endogenous or exogenous stimuli (infection and disease) and undergoes reconstitution in response to various antigens (Mikszta et al., 1999). In addition to TCR-mediated Ag recognition and pathogen clearance, our preceding work demonstrated an immunoregulatory function of TCR IR reconstitution in other immune cells in the hepatic immune microenvironment (Liang et al., 2018).

Despite all this knowledge, it remains unknown how various components of GM interact with intrahepatic TCR immune repertoire (IR). Next-generation sequencing (NSG) provides a tool for describing the features of TCR IR and the distribution of GM. In this study, we analyzed the GM profiles and TCR distribution using 16S rRNA gene sequencing (16S-seq) and bulk immune repertoire sequencing (IR-seq) to reveal the variations in GM and TCR IR during liver fibrogenesis, as well as associations between GM shift and TCR transformation. In addition, fecal microbial transplant (FMT) and TCRβ knockout (Tcrb^KO^) mouse models were employed to determine the functional link between GM and TCR IR in liver fibrosis.

## Materials and Methods

### Animal Models and Fecal Microbial Transplantation (FMT)

Wild-type C57BL/6 (WT) and Tcrb^KO^ mice were housed in pathogen-free animal rooms at the Animal Care Center of Xiamen University. All animal experiments were approved and conducted in accordance with the Guidelines of the Xiamen University Committee on Animal Care and Use (XMULAC20190061). For FMT, 8 to 12-week-old male mice were recolonized by FMT from WT mice. Mice were administered stool by oral gavage using animal feeding needles, given three times per week before CCl_4_ injection and once per week after CCl_4_ injection until the end point. To induce chronic liver fibrosis, mice were injected intraperitoneally with CCl_4_ (Sigma-Aldrich, St. Louis, MO, United States) at a dose of 1 μl/g body weight mixed with olive oil three times per week for 8 weeks. Animals were sacrificed 24 h after the last injection, and samples were harvested for subsequent analyses.

### DNA Extraction and 16S rRNA Sequencing

Cecal content was aseptically collected into 1.5 ml Eppendorf tubes. Microbial DNA was extracted using the HiPure stool DNA Kits (Magen, Guangzhou, China) according to the manufacturer’s protocols. The 16S rRNA V3-V4 region of the ribosomal RNA gene was amplified by PCR (94°C for 2 min, followed by 30 cycles at 98°C for 10 s, 62°C for 30 s, 68°C for 30 s, and a final extension at 68°C for 5 min). Amplicons were extracted from 2% agarose gels and purified using the AxyPrep DNA Gel Extraction Kit (Axygen Biosciences, Union City, CA, United States). Purified amplicons were pooled in equimolar, paired, and sequenced on the Novaseq 6000 platform (Illumina, San Diego, CA, United States) using the 2 × 250 bp paired-end protocol.

### Isolation of Intrahepatic Immunocytes and Flow Cytometry

Mouse intrahepatic immunocytes were isolated as previously described (Liang et al., 2018). The isolated immunocytes were incubated with fluorescently conjugated antibodies directed against mouse CD45 (FITC, I3/2.3, Cat#: 147709), B220 (PE, RA3-6B2, Cat#: 103207), CD3e (APC, 145-2C11, Cat#: 100312), TCRβ (FITC, clone: H57-597, Cat#: 109205), and TCRγδ (APC, clone: GL3, Cat#: 118115) from Biolegend (San Diego, CA, United States) for 20 min at 4°C in the dark. B cells (CD45^+^ and B220^+^), γδT cells (CD3e^+^ and TCRγδ^+^), and αβT cells (CD3e^+^ and TCRβ^+^) were obtained from intrahepatic immunocytes by flow cytometry. For cytokine detection, the selected cells were stained with antibodies against murine tumor necrosis factor α (TNF-α) and interleukin-17 (IL-17) from BD PharMingen (Mountain View, CA, United States) for 20 min at 4°C in the dark after incubation with permeabilization buffer. The stained cells were examined on a Beckman CytoFlex S (Beckman Coulter, Inc., Kraemer Boulevard Brea, CA, United States) and analyzed with the FlowJo software (version 10.0, TreeStar, Ashland, OR, United States).

### Construction, Sequencing, and Data Processing for TCR IR

The RNAprep Pure Cell/Bacteria Kit (Tiangen Biotech, Beijing, China) was used to extract total RNA from intrahepatic immune cells according to the manufacturer’s specifications. Then, 200 ng of total RNA was reverse-transcribed into cDNA using a Transcriptor First Strand cDNA Synthesis Kit (Roche Applied Science, Penzberg, Germany) on a C1000 Touch^TM^ Thermal Cycler (Bio-Rad Inc., Hercules, CA, United States). Two-round nested amplicon arm-PCR with specific primers was performed using 2 × SanTaq PCR Mix (Sangon Biotech, Shanghai, China) as previously described (Liang et al., 2018). Amplicons were extracted from 2% agarose gels and purified using the AxyPrep DNA Gel Extraction Kit (Axygen Biosciences, Union City, CA, United States). Purified amplicons were paired-end sequenced (PE250) on the Illumina platform according to standard protocols.

The clean data were harvested by filtering the low-quality sequences. The V, D, and J segments of the TCRβ consensus sequences were identified using the BLAST software (v2.2.25) in the international ImMunoGeneTics (IMGT) information system (IMGT)^1^ by a standard algorithm. The Gini index, Shannon diversity, and CDR3 clustering were calculated using the QIIME (version 1.9.1).

### Statistical Analysis

All statistical data were analyzed using the GraphPad Prism 8.0 Package (GraphPad Software, La Jolla, CA, United States). Data are presented as the mean ± standard deviation (SD). Student’s *t*-test or the Welch *t*-test was used to compare values between the two groups. The means of multiple groups were compared using one-way analysis of variance (ANOVA) followed by Tukey’s HSD test for mean separation. Statistical significance was accepted at *P* < 0.05. ^∗^*P* < 0.05; ^∗∗^*P* < 0.01; ^∗∗∗^*P* < 0.001.

Additional methods are provided in the Supplementary Material.

## Results

### GM Dysbiosis Occurs in Liver Fibrosis

**FIGURE 1:**
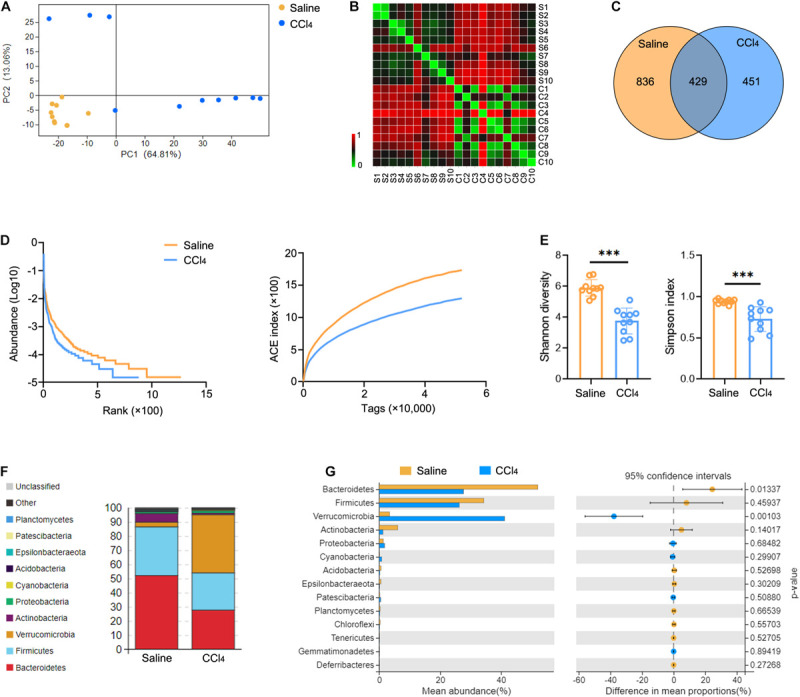
Liver fibrosis influences GM community and diversity. WT mice were treated three times weekly with CCl_4_ or 0.9% saline for 8 weeks. The 16S rRNA-seq data from cecal content were evaluated (*N* = 10). **(A,B)** Beta diversity determined by PCA **(A)** and Bray-Curtis distance **(B)**, showing closeness and distance between the two groups. **(C)** Venn analysis was performed to identify unique and common OTUs. **(D,E)** Alpha diversity determined by rank-abundance (left), ACE curve (right) **(D)**, Shannon diversity (left), and the Simpson index (right) **(E)** were used to measure the diversity of GM. **(F,G)** The composition **(F)** and abundance **(G)** of GM at the phylum taxonomic level. ***P < 0.001.

To characterize GM variations in the context of liver fibrosis, we constructed and sequenced 16S rRNA amplicon libraries from cecal content. Mice were administered 0.9% saline (Saline group) or CCl_4_ by intraperitoneal injection (CCl_4_ group) three times a week for 8 weeks. This induced distinct changes in microbiota composition due to fibrogenesis compared to controls as revealed by principal component analysis (PCA) (Figure 1A and Supplementary Table 1). The operational taxonomic units (OTUs) were observed by the Bray-Curtis distance analysis (Figure 1B and Supplementary Table 2). Interestingly, liver fibrosis caused an obvious decrease in OTUs (Figure 1C). We further measured microbial diversity using different methodologies (rank-abundance, ACE index, Shannon and Simpson index). The results showed that the diversity of GM was significantly lower in the CCl_4_ group than in the saline group (Figures 1D,E). Subsequently, we assessed the enrichment of specific bacterial communities in each group at the phylum level (Figure 1F and Supplementary Figure 1A). Bacteroidetes and Firmicutes were the most abundant in control mice, which was consistent with a previous study (Liu et al., 2016). Of note, liver fibrosis markedly increased Verrucomicrobia abundance while it decreased Bacteroidetes abundance (Figure 1G). To explore the potential function of GM in fibrogenesis, Phylogenetic Investigation of Communities by Reconstruction of Unobserved States (PICRUSt) was employed to predict the Kyoto Encyclopedia of Genes and Genomes (KEGG) pathways associated with components of the GM. Supplementary Figure 1B shows that a majority of functional biomarkers were enriched in metabolic pathways and less so in “Replication and repair,” “Membrane transport,” “Cell motility” as well as “Immune system” processes (Supplementary Table 3). Notably, liver fibrosis reinforced these pathway enrichments (Supplementary Figure 1C and Supplementary Table 4). These data suggest that liver fibrosis triggers GM dysbiosis, especially altering microbiota abundance and decreasing diversity.

### Liver Fibrosis Decreases Diversity of Intrahepatic TCR IR

**FIGURE 2:**
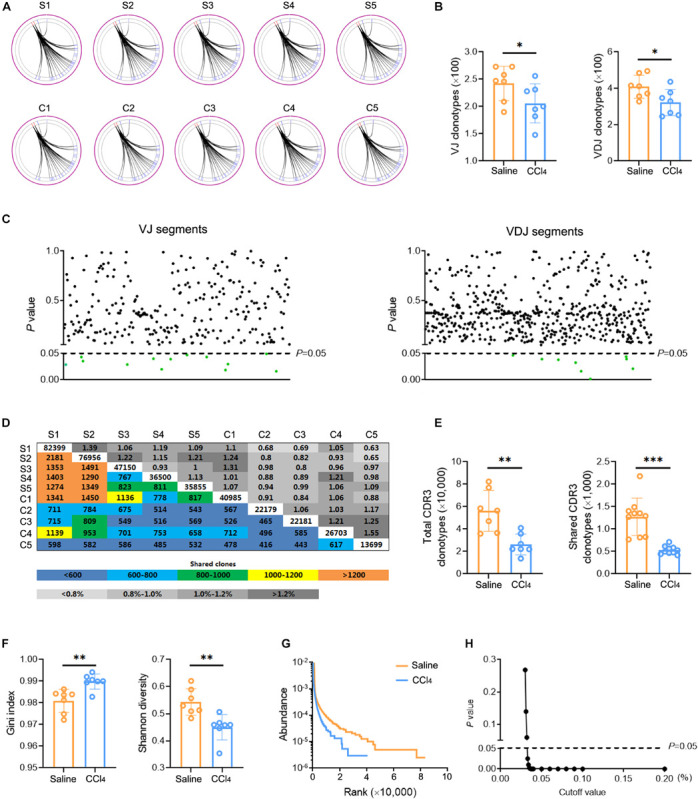
Fibrogenesis diminishes intrahepatic TCR diversity. WT mice were treated three times weekly with CCl_4_ or 0.9% saline for 8 weeks. TCR IR-seq data from intrahepatic isolated immune cells were evaluated (*N* = 5–7). **(A)** Circle diagram showing the composition of VJ segments. **(B)** Quantification of the composition of VJ segments (left) and VDJ segments (right). **(C)** The frequencies of VJ segments (left) and VDJ segments (right) that had significant differences (*P* < 0.05). Green color indicated downregulation. **(D)** Quantification (colored plots) and frequency (gray plots) of TCR CDR3 AA clones between two groups. Uncolored plots indicate the total clones per sample. **(E)** The total (left) and shared (right) CDR3 AA clones in each group. **(F)** The Gini index (left) and Shannon diversity (right) CDR3 AA clones in each group. **(G)** Rank-abundance showing CDR3 AA richness and evenness. **(H)** Comparison of clone frequencies for intrahepatic TCR CDR3 AA (dotted line indicates *P* = 0.05). The cut-off value depends on the clonal frequency exceeding a certain threshold. *P < 0.05; **P < 0.01; ***P < 0.001.

The changes in TCR IR in liver fibrosis were examined with bulk IR-seq. A total of 23 distinct V gene segments and 12 distinct J gene segments were identified from all samples (Supplementary Table 5). The most frequent V and J gene segments in the saline group were TRBV1 (37.58%) and TRBJ1-1 (15.02%). The most frequent V and J gene segments in the CCl_4_ group were TRBV1 (40.61%) and TRBJ2-1 (17.01%) (Supplementary Figure 2A). Based on the usage frequency of V and J gene segments, heat maps were generated as shown in Supplementary Figure 2B. The usage patterns of most V and J gene segments were similar between the two groups, but significant usage differences for four V gene segments were observed, including TRBV15, TRBV17, TRBV12-2, and TRBV23 (Supplementary Figure 2C). We also analyzed the composition of VJ segments and VDJ segments. A total of 276 distinct VJ segments and 537 distinct VDJ segments were identified from all samples (Supplementary Table 5). Strikingly, multiple VJ and VDJ segments were absent in the CCl_4_ group but were present in the saline group (Figures 2A,B). Of note, the usage of 13 VJ segments and nine VDJ segments in the CCl_4_ group was significantly lower than in the saline group (Figure 2C and Supplementary Figure 2D).

CDR3 amino acid (AA) clonotypes determine the diversity of the TCR IR. Thus, we investigated the effect of fibrogenesis on the diversity of CDR3 AA clonotypes. Overall, 384,216 distinct CDR3 AA clonotypes were identified from all samples (Supplementary Table 6). Although the overlap rate and length of CDR3 AA clonotypes were similar between the saline group and the CCl_4_ group (Figure 2D and Supplementary Figure 2E), CDR3 AA clones and shared CDR3 AA clones were lower in the CCl_4_ treatment group (Figure 2E). The Gini index and Shannon diversity revealed that liver fibrosis remarkably narrowed the diversity of CDR3 AA (Figure 2F). In addition, rank-abundance analysis demonstrated shrunken CDR3 AA richness and evenness in the CCl_4_ group compared to the saline group (Figure 2G). Although the total CDR3 AA clonal frequencies were nearly identical in the two groups, fibrogenesis caused obvious distinct usage in high-frequency CDR3 AA clonotypes (cut-off threshold >0.033%, *P* < 0.05) (Figure 2H). These data indicate that liver fibrosis can reset intrahepatic TCR IR, thereby lowering diversity.

### Intrahepatic TCR IR Diversity Is Associated With GM Diversity

**FIGURE 3:**
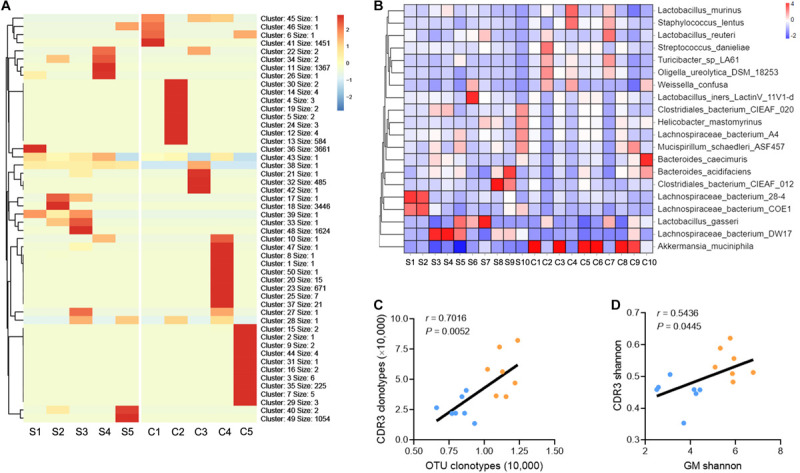
TCR IR distribution is associated with GM distribution. **(A)** Heat maps showing the hierarchical clustering of intrahepatic TCR CDR3 AA clusters (*N* = 5). **(B)** Heat maps showing the abundance of GM at the species taxonomic level (*N* = 10). **(C,D)** A plot of CDR3 AA clonotypes versus OUT clonotypes **(C)** and CDR3 AA Shannon diversity versus OTU Shannon diversity **(D)**. Pearson’s *r* values and the corresponding *P*-value are shown (*N* = 7).

The conclusion presented above indicate that liver fibrosis narrows the diversity of GM and intrahepatic TCR IR. To delineate the correction between the GM and TCR IR, a similar sequence clustering method for the CDR3 sequence was used in this work, and a total of 50 CDR3 clusters were identified (Supplementary Table 7). Clustering analysis revealed an aggregated expression of specific CDR3 clusters in liver fibrosis, and the expression of CDR3 clusters was spread and even in the saline group (Figure 3A). A similar phenomenon was observed in bacterial communities (at species level), and liver fibrosis obviously enhanced the abundance of *Akkermansia muciniphila* (*A. muciniphila*) and reduced the abundance of *Lachnospiraceae_bacterium_DW17* (Figure 3B, Supplementary Figure 3, and Supplementary Table 8). In addition, a significant positive association was observed between CDR3 AA clonotypes/Shannon diversity and OTU clonotypes/Shannon diversity (Figures 3C,D). Collectively, these data indicate that liver fibrosis induces the expression of the high-frequency CDR3 cluster and intestinal bacterial communities and that intrahepatic TCR IR diversity is associated with GM diversity.

### GM Participates in the Progression of Liver Fibrosis and Shapes Intrahepatic TCR IR

**FIGURE 4:**
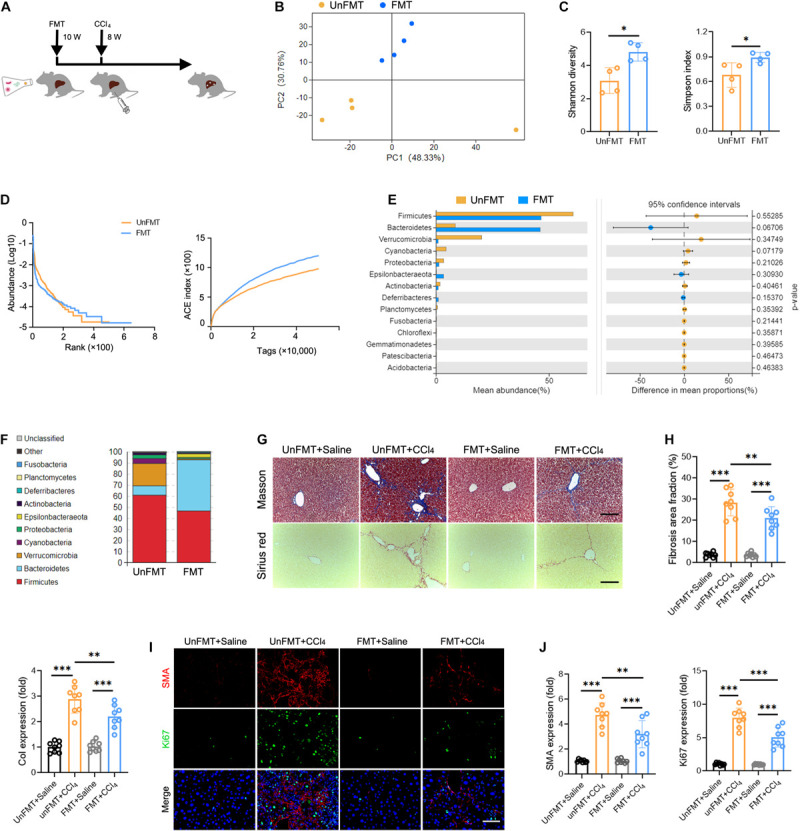
Gut microbiota (GM) regulates the development of liver fibrosis. **(A)** Experimental procedure of extracting FMT from WT mice in CCl_4_-treated mice (*N* = 4–8). **(B)** Beta diversity as determined by PCA, showing closeness between FMT mice and UnFMT mice. **(C,D)** Alpha diversity by Shannon diversity (left), Simpson index (right) **(C)**, rank-abundance (left), and ACE curve (right) **(D)**, showing the diversity of GM. **(E,F)** The composition **(E)** and the abundance **(F)** of GM at the phylum taxonomic level. **(G)** Representative images of Masson trichrome staining and Sirius red staining of FMT and UnFMT mice liver after CCl_4_ or 0.9% saline treatment for 8 weeks. Scale bar, 100 μm. **(H)** Quantification of fibrosis area fraction (left) and *Collagen-I* expression (right) as shown in **(G)**. **(I)** Liver sections were stained for SMA (red) and Ki67 (green) to identify the proliferative HSCs. Representative images are shown. Scale bar, 100 μm. **(J)** Quantification of SMA (left) and Ki67 (right) expression as shown in **(I)**. *P < 0.05; **P < 0.01; ***P < 0.001.

To determine whether GM can influence the progression of liver fibrosis, we performed fecal microbial transplantation (FMT). Fecal material from WT mice (FMT group) or 0.9% saline (UnFMT group) was transplanted by oral gavage three times a week. After 2 weeks, the mice were administered a CCl_4_ injection three times a week for 8 weeks (Figure 4A). The abundance and diversity of OTUs in FMT mice and UnFMT mice were also measured (Supplementary Table 9). As expected, an obvious change in microbiota composition (Figure 4B) and an increase in microbial diversity were observed in the FMT group (Figures 4C,D). Of note, FMT reversed the effect of fibrogenesis on the abundance of Verrucomicrobia and Bacteroidetes (Figures 4E,F). Subsequently, we assessed the progression of liver fibrosis in the context of GM changes. Masson staining and Sirius red assays revealed that FMT alleviated CCl_4_-induced liver fibrosis and *Collagen-I* expression (*Col1*) (Figures 4G,H). Consistently, a significant reduction in HSCs activation was observed in mice that received FMT compared with saline (UnFMT) (Figures 4I,J). Collectively, these results suggest that FMT delays liver fibrosis progression by modulating HSC activation.

**FIGURE 5:**
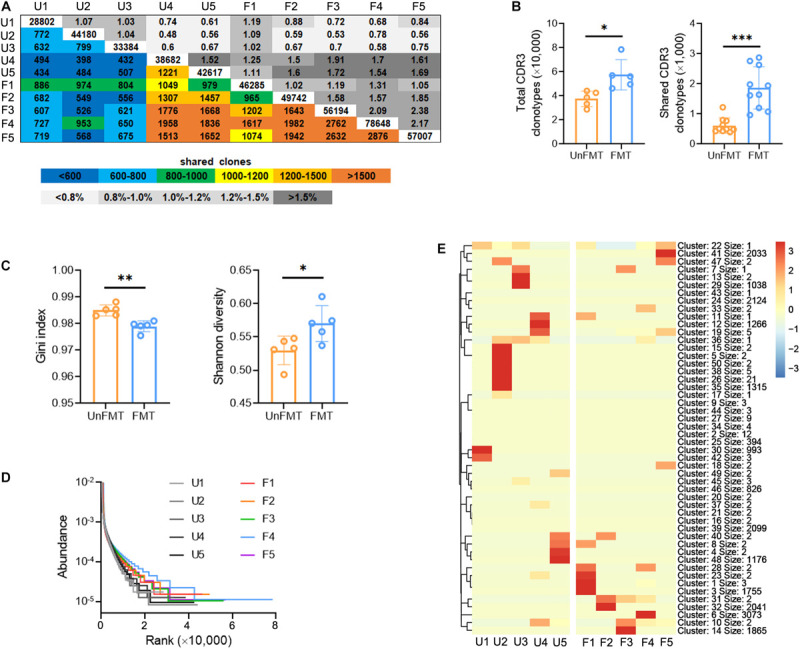
Fecal microbial transplant (FMT) shapes the intrahepatic TCR IR. **(A)** Quantification (colored plots) and frequency (gray plots) of intrahepatic TCR CDR3 AA clones from FMT mice and UnFMT mice. Uncolored plots indicate the total clones per sample (*N* = 5). **(B)** The total (left) and shared (right) CDR3 AA clones in each group. **(C)** The Gini index (left) and Shannon diversity (right) CDR3 AA clones in each group. **(D)** Rank-abundance showing CDR3 AA richness and evenness. **(E)** Heat maps showing the hierarchical clustering of CDR3 AA clusters. *P < 0.05; **P < 0.01; ***P < 0.001.

To investigate whether GM can influence intrahepatic TCR IR, we performed bulk IR-seq to assess variations in TCR IR. Interestingly, the distribution and abundance of V, J, VJ, and VDJ segments was not significantly different between FMT mice and UnFMT mice (Supplementary Figure 4 and Supplementary Table 10). However, it was noted that FMT ameliorated the inhibitory effect of fibrogenesis on CDR3 AA clonotypes (Figures 5A,B and Supplementary Table 11) and CDR3 AA diversity (as shown by the Gini index, Shannon diversity, and rank-abundance analysis) (Figures 5C,D). A similar sequence clustering analysis performed to detect CDR3 cluster changes, revealed that liver fibrosis led to the aggregation of specific CDR3 clusters, whereas FMT led to the diffusion of CDR3 clusters (Figure 5E and Supplementary Table 12). These data demonstrate that GM can shape the intrahepatic TCR IR.

### The GM/TCR IR/Immune Milieu Axis Modulates Liver Fibrosis

**FIGURE 6:**
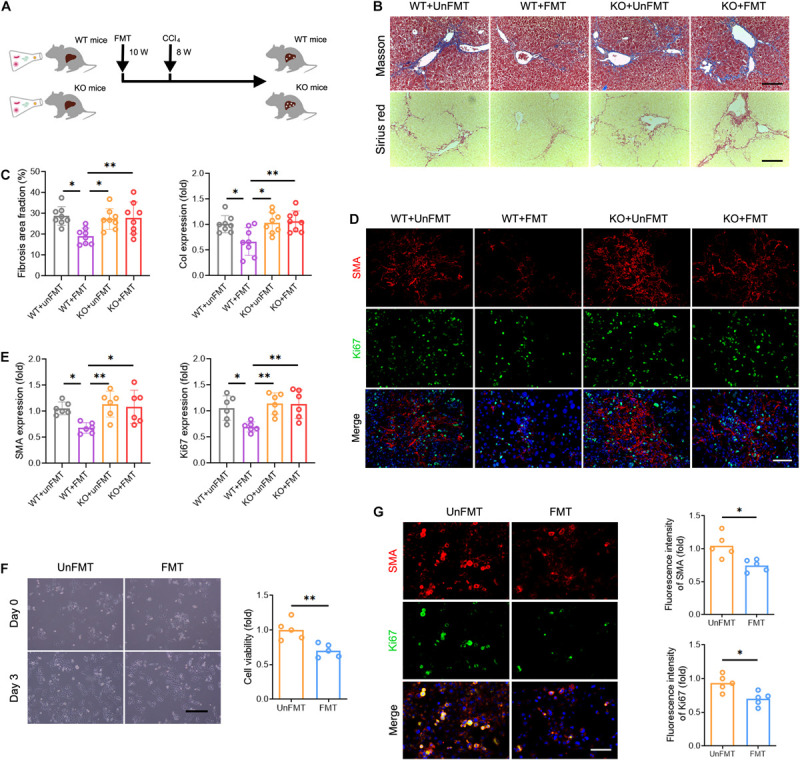
Deficiency of TCR IR abrogates the effects of GM on liver fibrosis. **(A)** Experimental procedure of extracting FMT and CCl_4_ treatment in WT mice and Tcrb^KO^ mice (*N* = 6–8). **(B)** Representative images of Masson staining and Sirius red staining of WT mouse and Tcrb^KO^ mouse livers after CCl_4_ treatment for 8 weeks. Scale bar, 100 μm. **(C)** Quantification of fibrotic area fraction (left) and *Collagen-I* expression (right) as shown in **(B)**. **(D)** Liver sections were stained for SMA (red) and Ki67 (green) to identify the proliferative HSCs. Representative images are shown. Scale bar, 100 μm. **(E)** Quantification of SMA (left) and Ki67 (right) expression as shown in **(D)**. **(F)** Representative images showing day 0 and day 3 co-cultures of primary HSCs (isolated from 6-week CCl_4_-treated liver) with intrahepatic immune cell (isolated from WT + UnFMT or WT + FMT mice liver after CCl_4_ treatment for 8 weeks) (left). Scale bar, 50 μm. Cell viability was estimated using the CCK-8 assay (right) after co-culture for 3 days (*N* = 5). **(G)** Immunofluorescence staining for SMA (red) and Ki67 (green) performed to identify the proliferating HSCs. Representative images are shown (left). Scale bar, 50 μm. Quantification of SMA (up) and Ki67 expression (down) (*N* = 5). *P < 0.05; **P < 0.01.

Thus far, we have demonstrated that GM regulates liver fibrosis and intrahepatic TCR IR. A key question yet to be answered is whether TCR IR mediates the effects of GM in liver fibrosis. To answer this question, Tcrb^KO^ mice were subjected to FMT derived from WT mice (Figure 6A). We observed no significant differences in appearance, architecture, fibrogenesis, and inflammation between WT mice and Tcrb^KO^ mice (Supplementary Figures 5A–C). The deletion of TCRβ in the Tcrb^KO^ mice was confirmed by immunofluorescence analysis and cytometry assay (Supplementary Figures 5D,E). TCR IR ablation reversed the suppressive effect of FMT on liver fibrogenesis and fibrogenic gene expression (Figures 6B,C). Notably, SMA and Ki67 expression restored HSC activation in Tcrb^KO^ mice that received FMT (Figures 6D,E). These results indicate that TCR IR mediates the effects of GM on liver fibrosis.

We then co-cultured primary HSCs (isolated from fibrotic liver) with or without T cells (isolated from normal liver) to verify whether T cells alone are responsible for HSC activation. Interestingly, we found no differences in HSC growth and proliferation among the different treatment groups, indicating that TCR IR is not the only driver of HSC activation (Supplementary Figure 6). Instead, other factors contribute to this process. To explore the regulatory role of the intrahepatic immune microenvironment on HSCs, co-cultures of mouse primary HSCs with intrahepatic immune cells, isolated from the WT + UnFMT group and the WT + FMT group, were performed for 3 days. A CCK-8 assay and Ki67 immunostaining were performed to confirm the HSC activation *in vitro*. Co-culture with intrahepatic immune cells isolated from the WT + FMT group notably inhibited HSC growth and proliferation compared with co-culture with intrahepatic immune cells isolated from the WT + UnFMT group (Figures 6F,G). Taken together, these results demonstrated that HSC activation is modulated by the intrahepatic immune microenvironment rather than by T cells alone.

**FIGURE 7:**
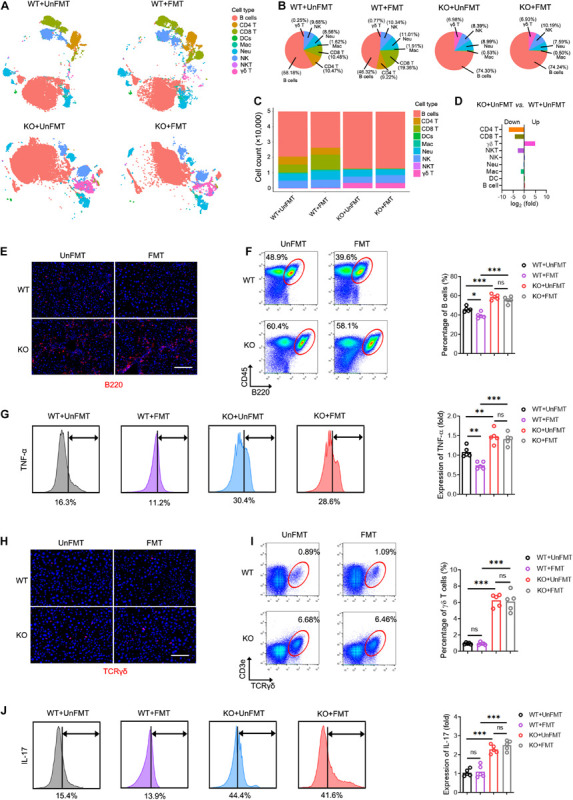
TCR IR influences intrahepatic immune microenvironment. **(A)** *t* distributed stochastic neighbor embedding (*t*SNE) clustering of intrahepatic immune cells from WT mice that received UnFMT, WT mice that received FMT, Tcrb^KO^ mice that received UnFMT, and Tcrb^KO^ mice that received FMT after 8-week CCl_4_ treatment, colored by immune cell type (*N* = 4–5). **(B,C)** The percentage **(B)** and the cell count **(C)** of each cell type. **(D)** The ratio [Tcrb^KO^ mice with UnFMT versus WT mice with UnFMT, log_2_(fold)] of distribution of each immune cell type. **(E)** Representative immunofluorescence image of B220 staining (red) in liver sections. Scale bar, 100 μm. **(F)** Representative cytometry plots of intrahepatic B cells (CD45^+^ and B220^+^) are shown. Quantification of the percentage of B cells. **(G)** Intrahepatic B cells were gated and used to test for expression of TNF-α. **(H)** Representative immunofluorescence image of TCRγδ (red) staining in liver sections. Scale bar, 100 μm. **(I)** Representative cytometry plots of intrahepatic γδT cells (CD3e^+^ and TCRγδ^+^) are shown. Quantification of the percentage of γδT cells. **(J)** Intrahepatic γδT cells were gated and used to test for expression of IL-17. *P < 0.05; **P < 0.01; ***P < 0.001.

Due to the compromised immune milieu in the fibrotic livers, we investigated whether TCRβ deficiency affects liver fibrosis by influencing the recruitment and activation of neighboring immunocyte subsets. We performed mass cytometry (CyTOF), targeting a global panel of 38 markers, and generated a detailed profile of the intrahepatic immune microenvironment (Supplementary Figure 7A). Characterization of the markers revealed nine distinct immunocyte subsets, including B cells, dendritic cells (DCs), macrophages, natural killer (NK) cells, neutrophils, CD4 T cells, CD8 T cells, γδ T cells, and natural killer T lymphocytes (NKT) in the liver (Figure 7A, Supplementary Figures 7B,C, and Supplementary Table 13). Of note, Tcrb^KO^ mice revealed abnormal B cell and γδ T cell expansion due to αβ T cell deficiency during liver fibrosis (Figures 7B–D). Compared with the UnFMT group, FMT gave rise to expanded CD8 T cells and shrunken B cells in WT mice liver (Figures 7E,F and Supplementary Figures 7D,E). Interestingly, expanded B cells with enhanced TNF-α levels (Figures 7E–G) and expanded γδ T cells with enhanced IL-17 levels were found in CCl_4_-treated Tcrb^KO^ mice with or without FMT (Figures 7H–J). However, FMT did not affect the distribution and activation of B cells and γδ T cells during liver fibrosis in Tcrb^KO^ mice (Figures 7E–J), suggesting that GM can shape intrahepatic immune cells by TCR IR. Collectively, these results suggest that FMT delays liver fibrosis progression by influencing TCR IR-mediated intrahepatic immune milieu.

## Discussion

Liver fibrosis may interfere with gut-bacterial interactions, leading to GM dysbiosis (Tilg et al., 2016; Tripathi et al., 2018b). Our data demonstrate that liver fibrogenesis is accompanied with reduced diversity of GM and decreased abundance of Bacteroidetes, an observation that was consistent with previous studies (Chen et al., 2011; Bajaj et al., 2014). Interestingly, liver fibrosis boosted *A. muciniphila* abundance. *A. muciniphila*, considered a promising probiotic, is known to have important value in improving host metabolic functions and immune responses (Derrien et al., 2017; Plovier et al., 2017; Zhang et al., 2019). Recent studies support a protective effect of *A. muciniphila* against hepatic injury, steatosis, and neutrophil infiltration (Wu et al., 2017; Kim et al., 2020). *A. muciniphila* also protects against ethanol-induced gut leakiness, enhanced mucus thickness, and tight-junction expression (Grander et al., 2018). In our study, CCl_4_ treatment enhanced *A. muciniphila* abundance, which may be explained as a feedback mechanism responding to hepatic injury. The breakdown of gut barrier integrity is mainly driven by gut inflammation and dysbiosis, both of which trigger bacterial translocation. Microorganisms and microorganism-derived molecules translocate to the liver through the portal system and interact with the intrahepatic immune microenvironment, causing inflammation and hepatic injury (Tripathi et al., 2018a). Although higher microbial diversity is known to modify the immune state, the “bridge” between GM and immune function is not entirely clear.

Identifying and tracking TCR IR by NSG provides a novel strategy to investigate the dynamics and distribution of all T cell clonotypes. TCR IR therefore serves as a “footprint” of immune status (Calis and Rosenberg, 2014). Emerging studies suggest that TCR IR participates in various liver diseases (including viral hepatitis, liver regeneration, hepatocellular carcinoma, primary sclerosing cholangitis, and alcoholic liver disease) (Chen et al., 2016; Huang et al., 2016; Liaskou et al., 2016; Jiang et al., 2018). In this study, we demonstrate the resetting of intrahepatic TCR IR in response to chronic liver injury, showing an intact characteristic of V, D, J, VJ, and VDJ segment usage and the distribution of CDR3 AA clonotypes during liver fibrosis. A lower diversity of CDR3 AA clonotypes along with diminished and centralized VJ and VDJ segments were observed during fibrogenesis. This is consistent with a recent work describing that liver remodeling leads to a remarkably lower TCR IR diversity and high-frequency TCR clonotypes expression (Liang et al., 2018).

Through a “gut-microbiome-liver” axis, the intestinal microbiome controls liver and immune functions (Woodhouse et al., 2018). Indeed, liver cirrhosis can be considered a microbiota-driven disorder, and a strong link between the GM and liver cirrhosis and its complications has been demonstrated (Adolph et al., 2018). The exact mechanism by which GM regulates liver fibrogenesis remains unclear. Previous studies have examined the metabolic role of GM in the liver. In the current study, the majority of functional biomarkers of GM were enriched in the metabolic pathways in liver fibrosis. However, the immune regulatory effects of GM in the liver should not be overlooked. The liver receives stimuli from the intestines that induce intrahepatic T cells recognition due to TCR IR reconstitution. Any change, therefore, in the GM composition will modify TCR IR reconstitution, thereby reshaping the intrahepatic immune microenvironment and the development of liver disease. In this study, changes in the distribution and diversity of GM correlated with changes in TCR IR during liver fibrosis. In addition, FMT restored the intrahepatic TCR IR diversity and improved liver fibrogenesis, while TCR IR ablation reversed the impact of FMT on fibrogenesis. These results suggest that intrahepatic TCR IR mediates the biological effect of GM in the development of liver fibrosis. Interestingly, although decreasing the GM by antibiotics or blocking their receptors results in decreased inflammatory and fibrogenic signaling in the liver (Fouts et al., 2012), the GM diversity plays an indispensable role in the maintenance of liver homeostasis. Furthermore, FMT did not alter the abundance of Verrucomicrobia and Bacteroidetes indicating that the normal balance in GM communities (both dominant microorganisms and inferior microorganisms) may prevent the development of liver fibrosis.

We also elucidated the potential mechanism by which TCR IR regulates HSC activation in a fibrotic liver. An interesting observation was that co-culturing HSCs with T cells isolated from WT mouse liver did not alter HSC activation and proliferation. In contrast, co-culturing HSCs with all immune cells isolated from FMT mouse liver suppressed HSC proliferation. This is in full agreement with our earlier conclusion that hepatic functions are regulated by many factors in the intrahepatic immune microenvironment and not solely by T cells (Liang et al., 2018). Accumulating studies indicate that HSCs receive many signals from hepatic immunocytes and in turn, produce many fibrotic mediators that broadcast the signals, thereby influencing liver fibrosis progression (Higashi et al., 2017; Tsuchida and Friedman, 2017). In this work, the intrahepatic immune cell landscape during liver fibrosis was examined using CyTOF. We found that FMT diminished B cells and expanded CD8^+^ T cells, thereby alleviating liver fibrogenesis as previously reported (Novobrantseva et al., 2005; Holt et al., 2006; Guidotti et al., 2015). Interestingly, TCR IR deficiency resulted in an aberrant intrahepatic immune microenvironment (expanded B cells with an enhanced TNF-α level, and expanded γδT cells with an enhanced IL-17 level) both in the Tcrb^KO^+UnFMT group and the Tcrb^KO^+FMT group during liver fibrogenesis. Indeed, B cell and γδT cell expansion are a normal characteristic of Tcrb^KO^ mice due to αβ T cell deficiency. However, FMT did not affect the distribution and activation of B cells and γδ T cells during liver fibrosis in Tcrb^KO^ mice, suggesting that TCR IR mediates the effects of GM on intrahepatic immune cells.

Liver injury leads to increases in the production of expanded T cell subsets from extrahepatic recruitment (Liang et al., 2018). Although the nature of the crosstalk between T cells and other immune cells is still unclear, recruited T cells pass through the liver vasculature, closely interacting with resident immunocytes, including intrahepatic γδT cells, NK cells, and Kupffer cells, on which they can directly exert their immunoregulatory effects (Heymann and Tacke, 2016; Liang et al., 2018). Conventionally, T cells are regulated by environmental stimuli via TCR and TCR-dependent environmental exposures (Xiao and Cai, 2017). The TCR IR patterns together with the specific TCR signaling associated with environmental exposures, jointly regulate the intrahepatic immune microenvironment. The reconstitution of TCR IR in the fibrotic liver observed in this study, provides novel insights into the immunosurveillance and immunoregulation of TCR IR that are synergistic with other immunocytes.

## Conclusion

**FIGURE 8:**
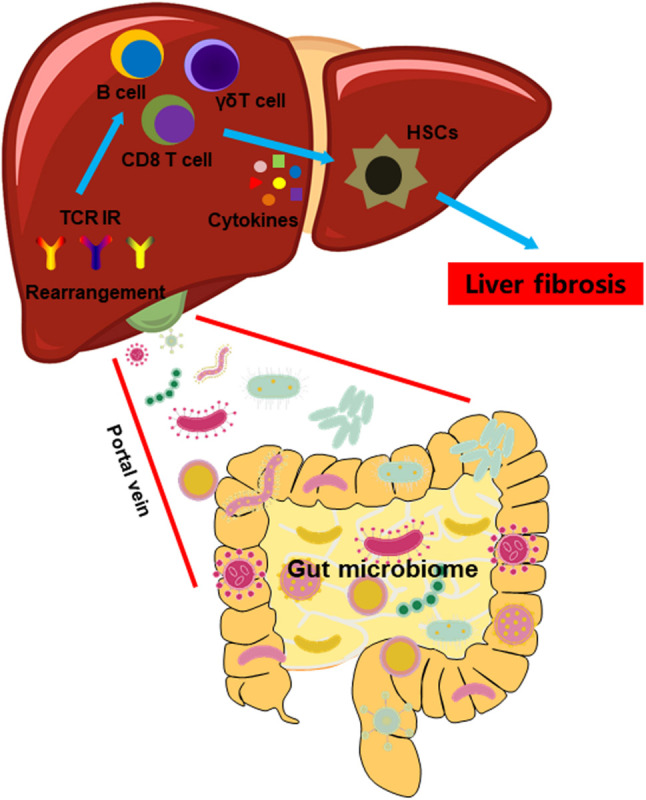
A model illustrating the impact of GM on the intrahepatic immune microenvironment in liver fibrosis.

We demonstrated a connection between GM, TCR IR, and the intrahepatic immune microenvironment. The dysbiosis of intestinal microbiota precipitated an unfavorable intrahepatic immune microenvironment, characterized by abnormal distribution and the activation of immune cell subsets due to TCR IR rearrangement (Figure 8). Thus, manipulation of GM may be an effective therapeutic opportunity to rebuild the intrahepatic immune microenvironment, hence improving liver fibrosis.

## Data Availability Statement

16S rRNA-seq and bulk IR-seq sequenced raw data were deposited under NGDC GSA database (https://bigd.big.ac.cn/gsa, accession code: CRA003301).

## Ethics Statement

The animal study was reviewed and approved by the Xiamen University Committee on Animal Care and Use.

## Author Contributions

KW conceived and designed the study, and wrote the manuscript. QL, MZ, WZ, PZ, and YC performed the experiments. QL and YH analyzed the CyTOF data. KW and QYL analyzed the 16S rRNA-seq data. KW and PX analyzed the bulk IR-seq data. All authors contributed to the article and approved the submitted version.

## Conflict of Interest

The authors declare that the research was conducted in the absence of any commercial or financial relationships that could be construed as a potential conflict of interest.

## Footnotes

1 http://www.imgt.org/
