# Supplementary material for: Gut Microbiome Contributes to Liver Fibrosis Impact on T Cell Receptor Immune Repertoire
Source: Front Microbiol. 2020 Nov 27;11:571847. doi: 10.3389/fmicb.2020.571847 (PMC7729130; doi:10.3389/fmicb.2020.571847)
Supplement: Supplementary file 15 [file Data_Sheet_1.docx]

**Gut microbiome shapes intrahepatic T cell receptor immune repertoire to influence liver fibrosis**

**Authors**

Qing Liang^1^, Meina Zhang^1^, Yudi Hu^1^, Wei Zhang^2^, Ping Zhu^3^, Yujie Chen^1^, Pengxin Xue^1^, Qiyuan Li^1^, and Kejia Wang^1*^

**Author affiliations**

^1^National Institute for Data Science in Health and Medicine, School of Medicine, Xiamen University, 361102 Xiamen, China

^2^Department of Pathology, The 971 Hospital of People’s Liberation Army Navy, 266071 Qingdao, China

^3^Department of Gynaecology and Obstetrics, The 971 Hospital of People’s Liberation Army Navy, 266071 Qingdao, China

***Correspondence to**

Professor Kejia Wang, School of Medicine, Xiamen University, South Xiang’an Road, 361102 Xiamen, China; wangkejia@xmu.edu.cn

**Supplementary Methods**

**Microbiome analysis**

Raw paired-end 16S rRNA reads were filtered according to the following rules using FASTP (Chen et al., 2018). The reads were subsequently merged as raw tags using FLSAH (Magoc and Salzberg, 2011) with a minimum overlap of 10 bp and mismatch error rates of 2%. Noisy sequences of raw tags were filtered by the QIIME pipeline (Caporaso et al., 2010) under specific filtering conditions to obtain high-quality clean tags. The effective tags were clustered into OTUs of ≥ 97% similarity using the UPARSE pipeline (Edgar, 2013). For community composition analysis, the representative sequences were classified into organisms by a naive Bayesian model using RDP classifier (version 2.2) (Wang et al., 2007) based on the SILVA database (version 132), Greengene database (version gg_13_5), UNITE database (version 8.0) or ITS2 database (version update_2015), with the confidence threshold values ranging from 0.8 to 1. Krona (Version 2.6) was used to visualize the abundance of each taxonomic unit. The KEGG pathway analysis of the OTUs was performed using PICRUSt (Langille et al., 2013).

**Quantitative real-time polymerase chain reaction (qPCR) assay**

Total RNA was isolated from liver tissues using TRIzol reagent (Vazyme, Nanjing, Jiangsu, China) following the manufacturer’s instructions. The quantity and quality of the RNA were determined using a spectrophotometer (Eppendorf BioPhotometer Plus, Eppendorf, Hamburg, Germany). Approximately 100 ng of RNA was reverse-transcribed to cDNA using a Transciptor First Strand cDNA Synthesis Kit (Vazyme). Real-time qPCR was conducted using SYBR Green Reagent (Vazyme) on a 7500 Fast Real Time PCR system (Applied Biosystems, CA, USA). The relative gene expression was normalized to the expression of GAPDH and calculated using 2^−ΔΔCt^ method.

**Histological and Immunofluorescence assays**

For histological and immunofluorescence assays, a haematoxylin and eosin (HE) staining kit (Solarbio Life Sciences, Beijing, China), Sirius red staining kit (Sigma-Aldrich, Shanghai, China) and Masson staining kit (Solarbio Life Sciences) were used per the manufacturer’s protocols. The details of the immunofluorescence staining can be found in our previous report (Liang et al., 2018). The following primary antibodies were used: CD8 (Abcam, clone: EPR21769, Cat#: ab217344), TCRβ (Abcam, clone: R73, Cat#: ab139367), TCRγδ (Abcam, clone: GL3, Cat#: ab231545), SMA (Abcam, clone: E184, Cat#: ab32575), and Ki67 (Abcam, clone: SP6, Cat#: ab16667). The liver fibrosis area fraction and immunofluorescence intensity were assessed in 10 areas using Image-Pro Plus 6.0 software (Media Cybernetics, Rockville, MD, USA).

**Isolation of HSCs and co-culture**

Primary HSCs were isolated as described previously with a few modifications (Oben et al., 2003). Briefly, the liver tissue was first perfused *in situ* with D-Hanks (Solarbio Life Sciences, Beijing, China) containing 25 U/ml heparin, then with D-Hanks containing pronase (0.05%, Solarbio Life Sciences), Ⅳ collagenase (1%, Solarbio Life Sciences) and DNase Ⅰ (1%, Solarbio Life Sciences) until the liver lost its firm texture. The liver was excised, placed in D-Hanks containing pronase, Ⅳ collagenase, and DNase Ⅰ at 37°C for 10 min. Next, the homogenate was filtered through a 75-μm gauge stainless steel mesh. The suspension was centrifuged (40 × g) for 3 min at 4°C, transferred to a new tube, then centrifuged (580 × g) for 8 min at 4°C. The supernatant was discarded and the pelleted cells were resuspended in 50% and 25% Percoll in D-Hanks containing 100 U/ml heparin (Solarbio Life Sciences), then centrifuged (900 × g) for 30 min at 4°C. The interface was gently aspirated, mixed with Dulbecco's Modified Eagle Medium (HyClone, Logan, UT, USA) and centrifuged at 400 × g for 10 min at 4°C. For co-culture experiments, the isolated HSCs were cultured in a 12-well plate (1 × 10^5^ cells/well) with RPMI 1640 medium (HyClone, Logan, UT, USA) containing penicillin and streptomycin, and 20% foetal bovine serum (HyClone) at 37°C for 24 h. Co-cultures were obtained by adding isolated intrahepatic T-cells (CD3e^+^ and TCRβ^+^, 1 × 10^4^ cells) to the HSC culture plates. RPMI 1640 medium with 5 ng/ml IL2, 20 ng/ml IL12, and 20 ng/ml IL18 (Carrier free, R&D Systems) was used for T-cell activation, and co-cultured cells were maintained for 3 days. The cell viability was measured using Cell Counting Kit-8 (CCK-8, Dojindo, Kumamoto, Japan). After 3 days of co-culture, a cell cycle assay was performed on a Beckman CytoFlex S (Beckman Coulter, Inc.) and FlowJo software (version 10.0) was used to analyse the data.

**CyTOF and data processing**

The isolated immune cells were stained with 0.5 μM cisplatin (diluted in prewarmed serum-free medium) on ice for 5 min and fixed with 1.6% paraformaldehyde for 10 min at room temperature in preparation for a viability test. Subsequently, the cells were incubated with Fc Receptor Blocking Solution (Fluidigm, South San Francisco, CA) for 10 min at room temperature and then stained with metal-conjugated antibody cocktail (38 antibodies in Table S14) overnight at 4°C. Afterwards, the cells were washed with cell staining buffer (PBS with 0.5% BSA and 0.02% sodium azide) twice. All data were acquired on a Helios mass cytometer (Fluidigm).

For CyTOF analysis, the raw fcs files were conducted as inputs of the function ‘cytof_exprs’ with ‘cytofAsinh’ as a transformation method using the Cytofkit (version 1.12.0) package in R software (version 3.6.3). A total of 50,000 cells were obtained from each sample and merged into an integrated MFI matrix. The FlowSOM clustering algorithm was then performed to cluster different types of cells with FlowSOM_k = 20 using the function ‘cluster_FlowSOM_x’. Finally, tSNE was performed to reduce the dimensions and visualize the data.

**Supplementary Figures**


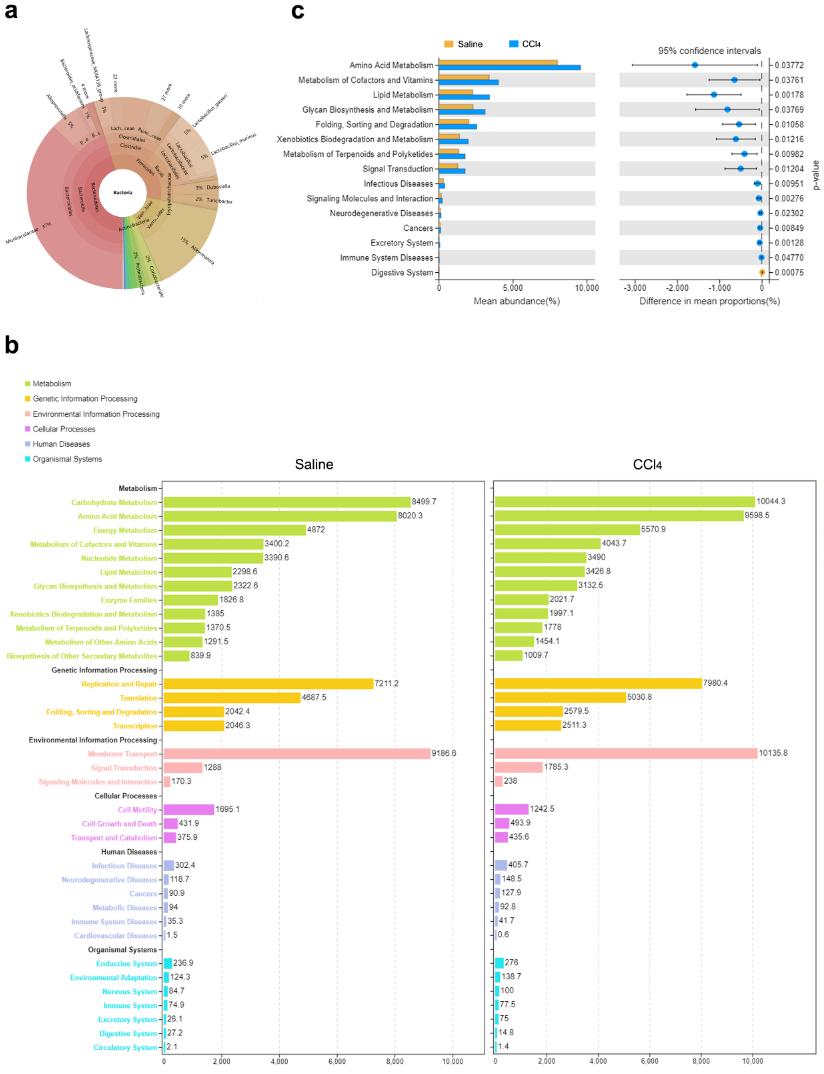


**Figure S1.** GM influences diverse signalling pathways in liver fibrosis. (a) The abundance of each taxonomic unit visualized using Krona. (b) PICRUSt analysis of the inferred OTUs for pathway enrichment. (c) Comparison of functional differences between the two groups.


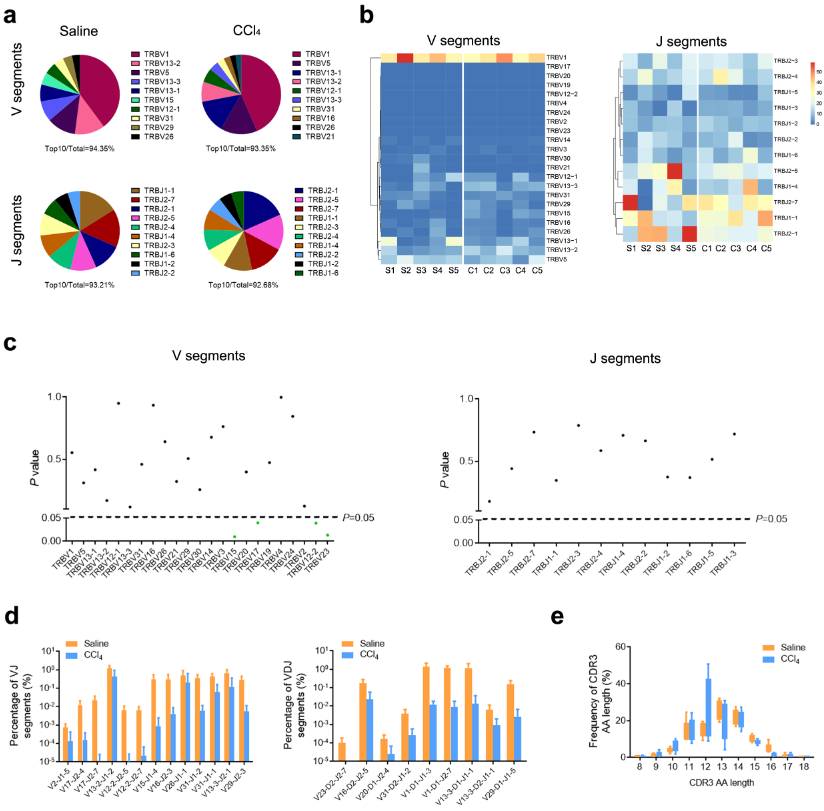


**Figure S2.** Fibrogenesis results in intrahepatic TCR IR reconstitution. (a) The distribution of the top 10 V and J gene segments in each group. (b) Heatmaps of hierarchical clustering of V and J gene frequencies. (c) The frequency [disparities](http://dict.youdao.com/w/discrepancy/#keyfrom=E2Ctranslation) in V segments and J segments between the two groups (dotted line indicates *P*=0.05, green colour indicates downregulation). (d) The abundance of differential VJ segments and VDJ segments as shown in Figure 2C. (e) The frequency distribution of CDR3 AA length between the two groups.


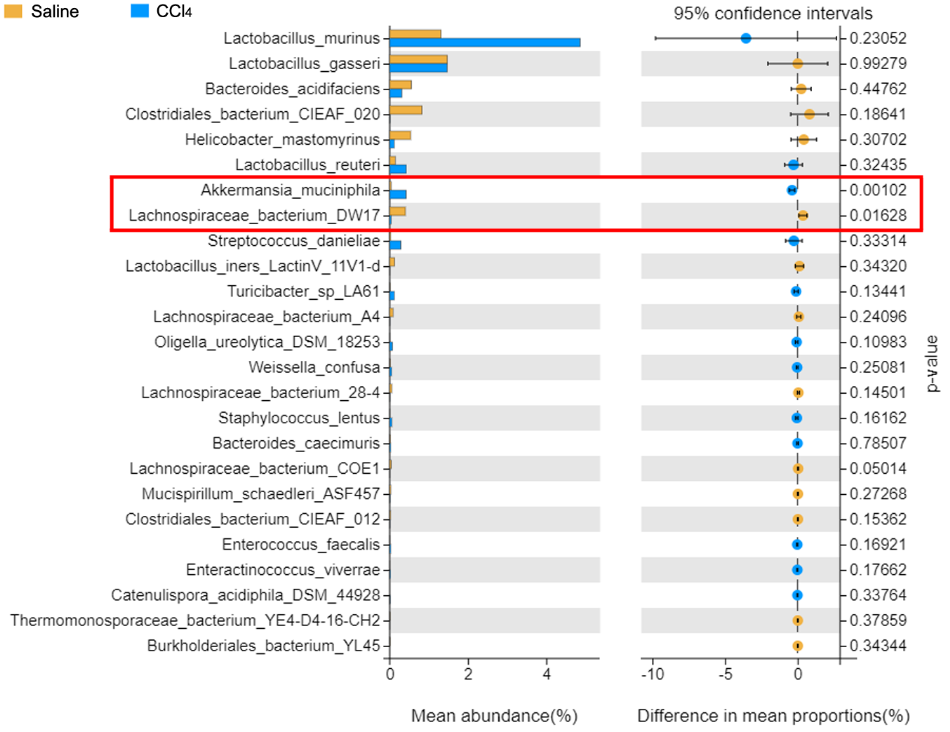


**Figure S3.** Comparison of the abundance of GM at the species taxonomic level between the Saline and CCl_4_ groups.


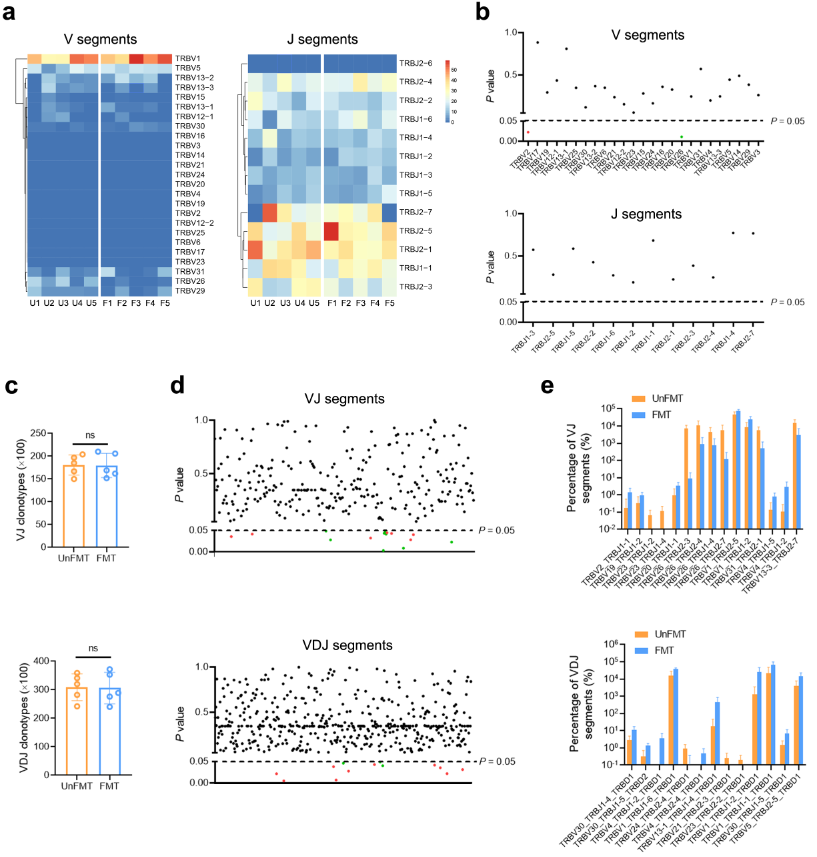


**Figure S4.** Fibrogenesis results in intrahepatic TCR IR reconstitution. (a) Heatmaps of hierarchical clustering of V and J gene frequencies between UnFMT mice and FMT mice. (b) The frequency disparities in V segments and J segments (dotted line indicates *P*=0.05, green colour indicates downregulation, red colour indicates upregulation). (c) Quantification of the composition of VJ segments (up) and VDJ segments (down). (d) The frequency [disparities](http://dict.youdao.com/w/discrepancy/#keyfrom=E2Ctranslation) in VJ segments and VDJ segments (dotted line indicates *P*=0.05, green colour indicates downregulation, red colour indicates upregulation). (e) The abundance of differential VJ segments and VDJ segments as shown in (d).


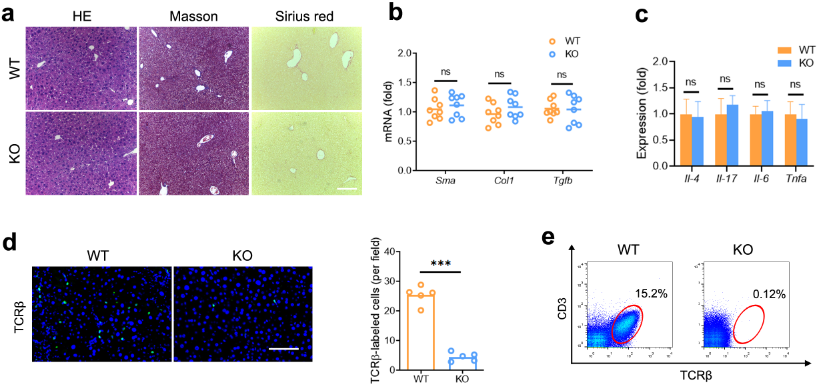


**Figure S5.** TCR IR does not affect liver development. (a) Representative HE, Masson and Sirius red staining images of WT and Tcrb^KO^ mouse liver before CCl_4_ treatment (N = 5-8). Scale bar, 100 μm. (b, c) qPCR results showing the mRNA expression of *Sma*, *Collagen-Ι*, *Tgfb*, *Il-4*, *Il-17*, *Il-6* and *Tnfa* in the liver. (d) Representative immunofluorescence image of TCRβ staining (green) in liver sections (left). Scale bar, 100 μm. Quantification of TCRβ-labelled cells (right). (e) Representative flow cytometry plot for intrahepatic αβT from WT and Tcrb^KO^ mice.


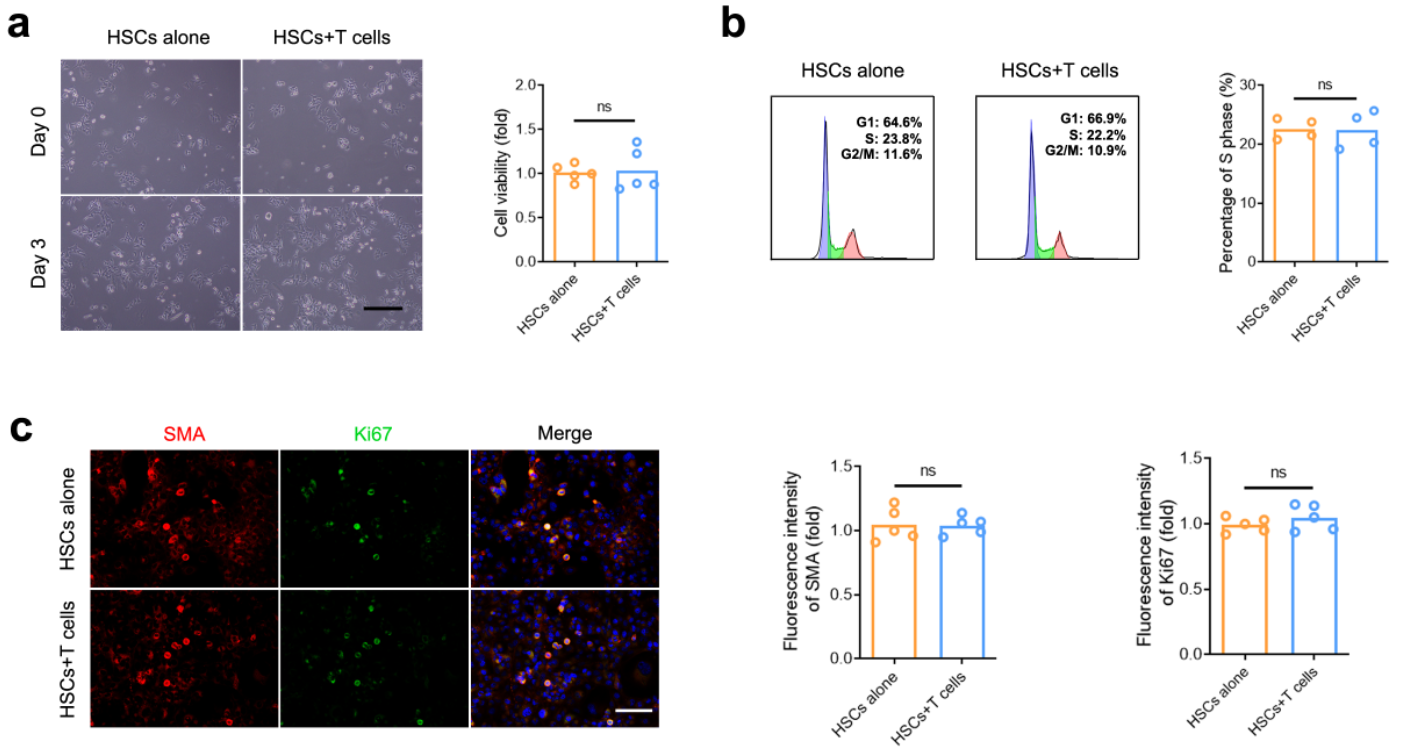


**Figure S6.** T cells alone do not regulate HSC activation. (a) Representative images showing day 0 and day 3 co-cultures of primary HSCs (isolated from 6-weeks CCl_4_-treated liver) with or without intrahepatic T cells (isolated from WT mouse liver) (left). Scale bar, 50 μm. Cell viability was estimated using the CCK-8 assay (right) (N = 5). (b) Cell cycle analysis for HSCs after co-culture for 3 days (N = 4). (c) Immunofluorescence staining for SMA (red) and Ki67 (green) performed to identify the proliferating HSCs. Representative images are shown (left). Scale bar, 50 μm. Quantification of SMA (middle) and Ki67 expression (right) (N = 5).


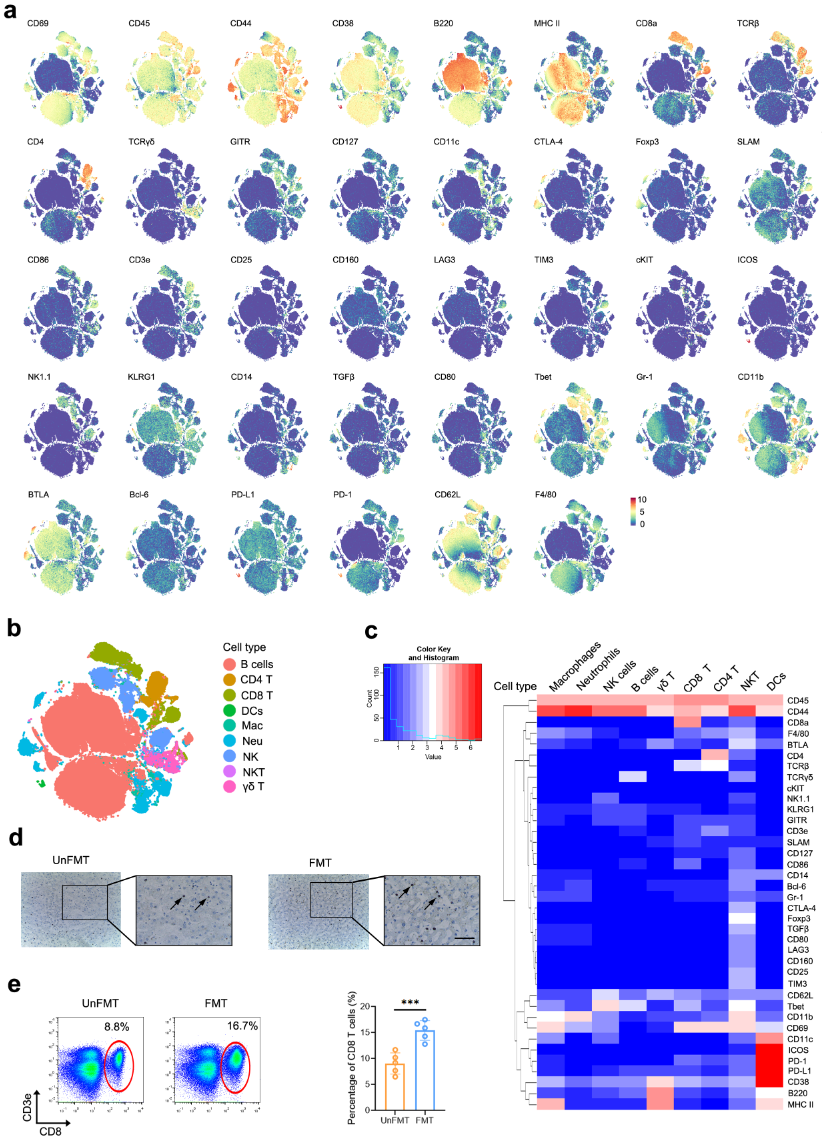


**Figure S7.** CyTOF analysis of intrahepatic immune cells. (a) *t*SNE clustering of 200,000 immune cells in liver tissues from WT mice that received unFMT, WT mice that received FMT, Tcrb^KO^ mice that received UnFMT and Tcrb^KO^ mice that received FMT after 8-week CCl_4_ treatment. Colours represent the expression values of the indicated markers (N = 4-5). (b) Nine immunocyte subsets identified by *t*SNE. (c) A heatmap showing the expression of markers of each cell type. (d) Representative images of immunohistochemistry results of intrahepatic CD8 T cells in the WT+UnFMT and WT+FMT groups. Scale bar, 100 μm. (e) Representative plots of intrahepatic CD8 T cells (CD3e^+^ and CD8^+^) are shown. Quantification of the percentage of CD8 T cells.

**References**

Caporaso, J.G., Kuczynski, J., Stombaugh, J., Bittinger, K., Bushman, F.D., Costello, E.K., Fierer, N., Pena, A.G., Goodrich, J.K., Gordon, J.I., Huttley, G.A., Kelley, S.T., Knights, D., Koenig, J.E., Ley, R.E., Lozupone, C.A., Mcdonald, D., Muegge, B.D., Pirrung, M., Reeder, J., Sevinsky, J.R., Turnbaugh, P.J., Walters, W.A., Widmann, J., Yatsunenko, T., Zaneveld, J., and Knight, R. (2010). QIIME allows analysis of high-throughput community sequencing data. *Nat Methods* 7**,** 335-336.

Chen, S., Zhou, Y., Chen, Y., and Gu, J. (2018). fastp: an ultra-fast all-in-one FASTQ preprocessor. *Bioinformatics* 34**,** i884-i890.

Edgar, R.C. (2013). UPARSE: highly accurate OTU sequences from microbial amplicon reads. *Nat Methods* 10**,** 996-998.

Langille, M.G., Zaneveld, J., Caporaso, J.G., Mcdonald, D., Knights, D., Reyes, J.A., Clemente, J.C., Burkepile, D.E., Vega Thurber, R.L., Knight, R., Beiko, R.G., and Huttenhower, C. (2013). Predictive functional profiling of microbial communities using 16S rRNA marker gene sequences. *Nat Biotechnol* 31**,** 814-821.

Liang, Q., Liu, Z., Zhu, C., Wang, B., Liu, X., Yang, Y., Lv, X., Mu, H., and Wang, K. (2018). Intrahepatic T cell receptor beta immune repertoire is essential for liver regeneration. *Hepatology*.

Magoc, T., and Salzberg, S.L. (2011). FLASH: fast length adjustment of short reads to improve genome assemblies. *Bioinformatics* 27**,** 2957-2963.

Oben, J.A., Yang, S., Lin, H., Ono, M., and Diehl, A.M. (2003). Acetylcholine promotes the proliferation and collagen gene expression of myofibroblastic hepatic stellate cells. *Biochem Biophys Res Commun* 300**,** 172-177.

Wang, Q., Garrity, G.M., Tiedje, J.M., and Cole, J.R. (2007). Naive Bayesian classifier for rapid assignment of rRNA sequences into the new bacterial taxonomy. *Appl Environ Microbiol* 73**,** 5261-5267.
